# Stabilisation of Fe_2_O_3_-rich Perovskite Nanophase in Epitaxial Rare-earth Doped BiFeO_3_ Films

**DOI:** 10.1038/srep13066

**Published:** 2015-08-14

**Authors:** Huairuo Zhang, Ian M. Reaney, Daniel M. Marincel, Susan Trolier-McKinstry, Quentin M. Ramasse, Ian MacLaren, Scott D. Findlay, Robert D. Fraleigh, Ian M. Ross, Shunbo Hu, Wei Ren, W. Mark Rainforth

**Affiliations:** 1 Department of Materials Science & Engineering, University of Sheffield, Sheffield S1 3JD, UK; 2 Department of Materials Science and Engineering and Materials Research Institute, The Pennsylvania State University, University Park, PA 16802, USA; 3 SuperSTEM Laboratory, STFC Daresbury Campus, Daresbury WA4 4AD, UK; 4 School of Physics and Astronomy, University of Glasgow, Glasgow G12 8QQ, UK; 5 School of Physics and Astronomy, Monash University, Clayton, Victoria 3800, Australia; 6 Department of Physics, The Pennsylvania State University, University Park, PA 16802, USA; 7 Kroto Centre for High Resolution Imaging & Analysis, Department of Electronic and Electric Engineering, University of Sheffield, Sheffield S1 3JD, UK; 8 Department of Physics, International Center for Quantum and Molecular Structures, and Materials Genome Institute, Shanghai University, Shanghai 200444, China

## Abstract

Researchers have demonstrated that BiFeO_3_ exhibits ferroelectric hysteresis but none have shown a strong ferromagnetic response in either bulk or thin film without significant structural or compositional modification. When remanent magnetisations are observed in BiFeO_3_ based thin films, iron oxide second phases are often detected. Using aberration-corrected scanning transmission electron microscopy, atomic resolution electron energy loss spectrum-mapping and quantitative energy dispersive X-ray spectroscopy analysis, we reveal the existence of a new Fe_2_O_3_-rich perovskite nanophase, with an approximate formula (Fe_0.6_Bi_0.25_Nd_0.15_)^3+^ Fe^3+^O_3_, formed within epitaxial Ti and Nd doped BiFeO_3_ perovskite films grown by pulsed laser deposition. The incorporation of Nd and Bi ions on the A-site and coherent growth with the matrix stabilise the Fe_2_O_3_-rich perovskite phase and preliminary density functional theory calculations suggest that it should have a ferrimagnetic response. Perovskite-structured Fe_2_O_3_ has been reported previously but never conclusively proven when fabricated at high-pressure high-temperature. This work suggests the incorporation of large A-site species may help stabilise perovskite-structured Fe_2_O_3_. This finding is therefore significant not only to the thin film but also to the high-pressure community.

Many of the initial problems encountered with the development of BiFeO_3_ as a ferroelectric or magnetoelectric material relate to its large conductivity and its high T_C_^1^,^2^,^3^,^4^,^5^,^6^. Although there is controversy as to whether undoped BiFeO_3_ is *p* or *n*-type, it has been reported that acceptor doping increases conductivity^7^ whereas donor doping reduces conductivity^8^ particularly if rare earth ions are used to partially substitute for Bi, thereby simultaneously lowering T_C_^9^. However, as the rare earth ion concentration increases, either the solubility limit is breached (for rare earth ionic radii <1.15 Å) or a PbZrO_3_-like antipolar phase is stabilised^9^,^10^,^11^. This aspect of the crystal chemistry of rare earth doped BiFeO_3_ ceramics has impeded development of bulk materials that can be switched at applied fields smaller than 10 kV/cm. Consequently, there has been significant attention placed on the development of rare earth doped BiFeO_3_ thin films where large fields may be readily applied and the breakdown strengths are typically enhanced with respect to bulk materials. Of particular note is the work of Takeuchi and co-workers^12^ and Nagarajan and co-workers^13^ who have developed a wide range of rare earth doped films and solid solutions using conventional and combinatorial synthesis via pulsed laser deposition (PLD). These authors reported ferroelectric switching for Sm doped systems for compositions with T_C_ ≈ 350 °C (x = 0.14) which appear to have a mixed ferroelectric/antiferroelectric phase assemblage, unlike the single phase PbZrO_3_-like Nd doped bulk compositions reported by Reaney and co-workers^9^,^10^,^11^.

A further issue encountered in BiFeO_3_ thin films is the appearance of iron-oxides which often result in unusually high remanent magnetisation^5^,^6^. There are many possible iron-oxide structures that may form during fabrication of BiFeO_3_ thin films. For example, corundum-structured Fe_2_O_3_ is commonly observed but in principle other phases such as Fe_3_O_4_ or FeO are possible^5^,^6^. In addition, there are several high temperature/pressure polymorphs of Fe_2_O_3_^14^. These include a disputed orthorhombic Fe^2+^Fe^4+^O_3_ perovskite structure with FeO_6_ octahedral corner-sharing alluded to by the authors of ref. 15, which some researchers suggest is an orthorhombic Rh_2_O_3_-II type Fe^3+^_2_O_3_ structure with FeO_6_ octahedral edge-sharing^16^,^17^.

In this work, we report an unusual Fe_2_O_3_-rich perovskite nanophase with an approximate formula, (Fe_0.6_Bi_0.25_Nd_0.15_)^3+^Fe^3+^O_3_ within epitaxial BiFeO_3_ films doped with Nd and Ti. The Fe_2_O_3_-rich perovskite phase forms principally as needles or laths ~10–20 nm wide perpendicular to the substrate in the upper regions of the film and is stabilised by Nd and Bi on the A-site. Stabilisation of Fe_2_O_3_-rich perovskite, even as a coherent second phase, suggests that epitaxial films of this composition could be fabricated on substrates such as DyScO_3_ which have a similar prototype perovskite lattice parameter *(a*_*0*_ = 0.394 nm) to the Nd doped BiFeO_3_ matrix (0.39 nm)^18^. The properties of Fe_2_O_3_-rich perovskite are not known, but the small ion size of Fe^3+^ in the pseudo-cuboctahedral cage of the perovskite lattice and the complex superexchange between A and B-site Fe^3+^ should make for intriguing fundamental physical properties. Preliminary density functional theory (DFT) calculations have already suggested that the Fe_2_O_3_-rich perovskite phase is ferrimagnetic.

## Results and Discussion

**Figure 1:**
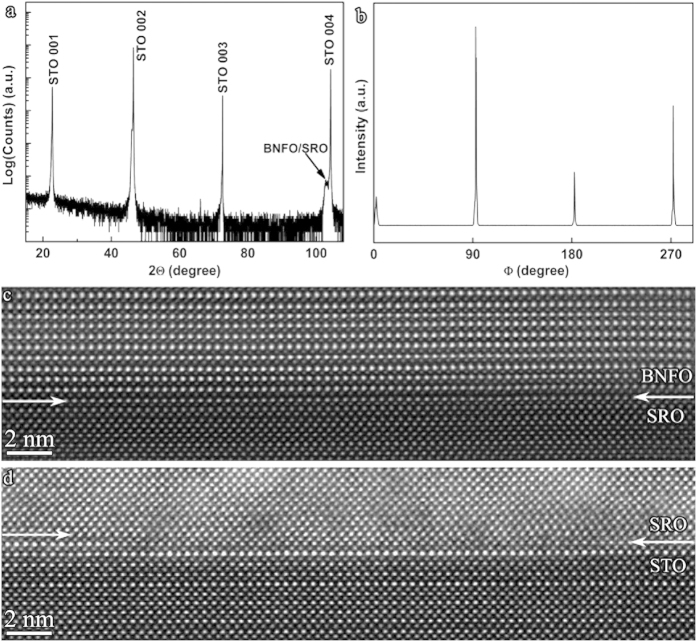
X-ray diffraction and atomic resolution HAADF imaging analyses of the BNFO/SRO/STO film. (**a**) *θ*–2*θ* scan and (**b**) Phi scans of BNFO/SRO/STO pseudocubic (002) peak. (**c**) and (**d**) Atomic resolution HAADF images showing the sharp interfaces of BNFO/SRO and SRO/STO, respectively.

**Figure 2:**
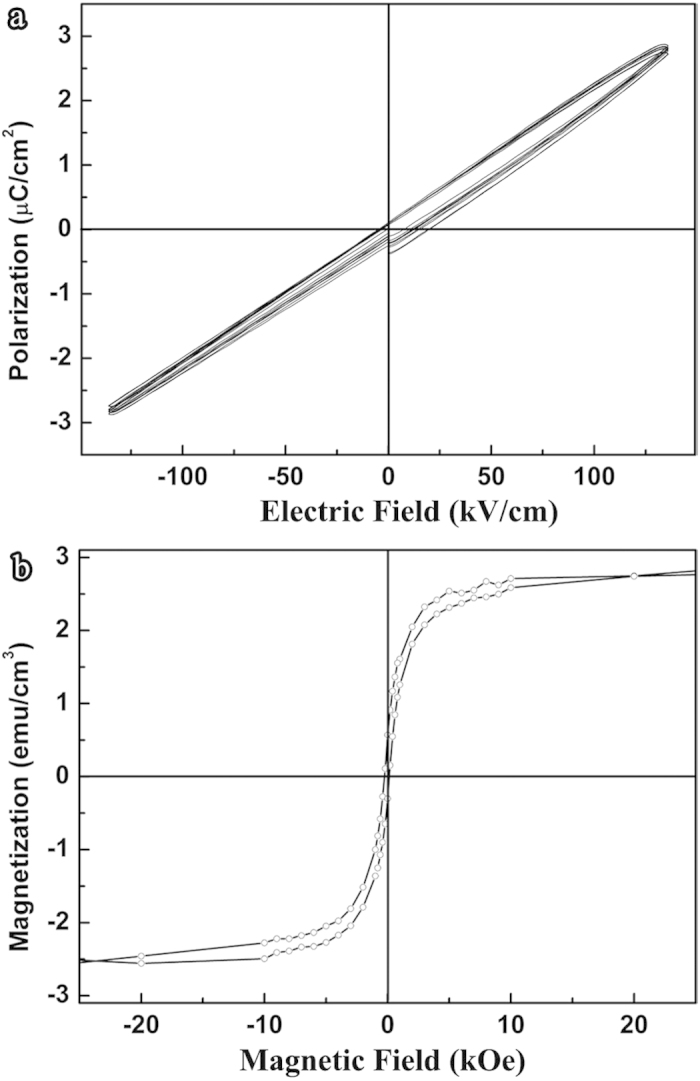
Polarization and magnetization measurements at 300 K. (**a**) Polarization-electric field (*P* - *E*) hysteresis loops showing linear dielectric behaviour. (**b**) Magnetization-magnetic field (perpendicular to the film) (*M* - *H*) hysteresis loop showing a weak ferromagnetic response.

XRD analysis shows that the BNFO film is epitaxial with the pseudocubic <001> orientations parallel to those of the SRO/STO substrate. The *θ*–2*θ* scan reveals a very small splitting in the high angle diffraction pseudocubic (004) peak of BNFO film and SRO/STO substrate (Fig. 1a). Phi scans of BNFO/SRO/STO overlapped pseudocubic (002) peak show sharp peaks (Fig. 1b), suggesting a high quality epitaxial film which is evidenced by the atomic resolution HAADF imaging analysis on the BNFO/SRO and SRO/STO interfaces (Fig. 1c,d). Polarization vs. electric field (*P-E*) (Fig. 2a) and magnetization vs. magnetic field (*M-H*) (Fig. 2b) measurements show that the BNFO film exhibits a linear dielectric and a weak ferromagnetic response.

**Figure 3:**
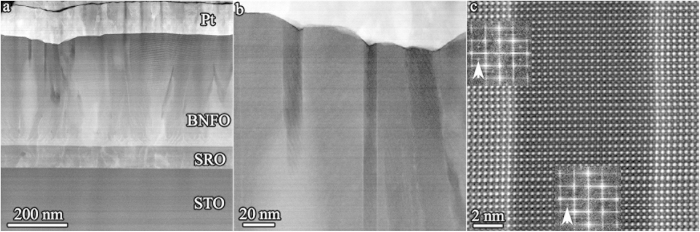
Fe-rich perovskite phase in BNFO film. (**a**) HAADF cross-sectional image of a (Bi_0.75_Nd_0.25_)(Fe_0.97_Ti_0.03_)O_3_ film grown on SrRuO_3_/SrTiO_3_ substrate showing the dark needle-shaped Fe-rich phase in the upper relaxed region of the film. (**b**) Enlarged image showing the vertical needle structure. (**c**) Atomic resolution image illustrating that the phase is fully coherent with the matrix film and the cations occupy similar positions as the BNFO perovskite matrix. Insets are the Fourier transform diffractograms from the corresponding areas, which contain ½{*oeo*} superstructure reflections due to in-phase octahedral tilting (where ‘*o*’ represents odd index number, and ‘*e*’ even index number).

Figure 3a,b show HAADF cross-sectional images of a BNFO epitaxial film deposited on SRO/STO. Regions of dark contrast 10–20 nm wide are observed that run perpendicular to the substrate in the upper relaxed region of the film. Atomic resolution HAADF images acquired on a double aberration corrected microscope JEM-Z3100F-R005 STEM/TEM operated at 300 keV demonstrate that the dark region is coherent with the matrix, and has the same cation structure as the BNFO perovskite matrix (Fig. 3c). Diffractograms obtained by Fourier transform of Fig. 3c revealed that the dark phase exhibits ½(*oeo*) superlattice reflections associated with in-phase tilting of the O-octahedra, similar to the matrix and consistent with *Pnma* symmetry (*a*^−^*b*^+^*a*^−^ octahedral tilting system)^11^. Quantitative EDS analysis revealed that the dark phase has an approximate cation composition: 80 at.% Fe, 12.5 at.% Bi and 7.5 at.% Nd (Ti was omitted due to the very weak Ti peaks which were barely above noise levels). Based on EDS, HAADF images and Fourier transform data, the dark phase appears to be an Fe_2_O_3_-rich perovskite phase with a likely formula (Fe_0.6_Bi_0.25_Nd_0.15_)FeO_3_. However, further proof is required to determine whether Fe resides on both the A- and B-sites. Consequently, further detailed analysis of the perovskite-like dark phase was performed on a Nion UltraSTEM microscope operated at 100 keV.

**Figure 4:**
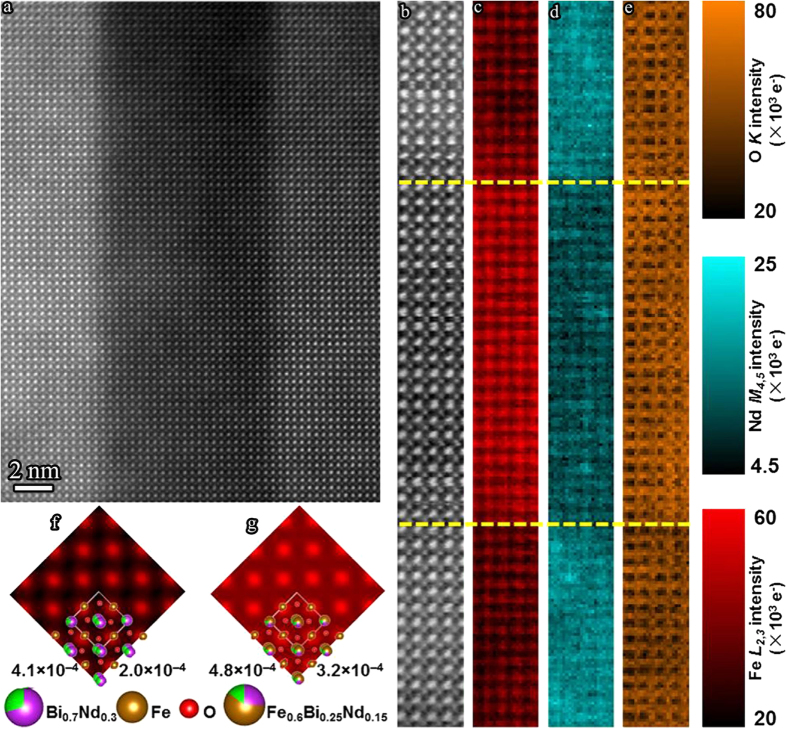
Atomic resolution EELS spectrum-imaging analysis. (**a**) HAADF image showing a region containing the Fe-rich perovskite phase. (**b**) HAADF image acquired simultaneously with the EELS spectrum-imaging (scanning direction was 90° rotated compared to Fig. 4a). (**c**) Fe-*L*_2,3_ map. (**d**) Nd-*M*_4,5_ map. (**e**) O-*K* map. Yellow dotted lines are added as a guide to the eye to mark the position of the interfaces between the BNFO matrix and Fe-rich phase. (**f**,**g**) Simulated Fe-*L*_2,3_ maps with overlapped (Bi_0.7_Nd_0.3_)FeO_3_ and (Fe_0.6_Bi_0.25_Nd_0.15_)FeO_3_ structure models, respectively, displayed on a common coloured contrast scale. The maximum and mean signal is given below each simulated image.

Figure 4a shows a HAADF image containing the Fe-rich perovskite phase. A nearby region was sampled for atomic resolution EELS spectrum-imaging analysis (Fig. 4b). Figure 4c–e are the corresponding Fe-*L*_2,3_ map, Nd-*M*_4,5_ map and O-*K* map, respectively, which confirm that the phase is Fe-rich and Nd-deficient. The Fe-*L*_2,3_ map indicates that the Fe signal is largest for the B-site columns, with a qualitatively stronger Fe signal in the Fe-rich phase than in the BNFO matrix but with no apparent Fe signal peak on the A-site of the Fe-rich phase. To understand this observation, Fe-*L*_2,3_ maps were simulated using the multislice method based on a frozen phonon model^19^,^20^,^21^. Simulations of the Fe-*L*_2,3_ map with structural models of (Bi_0.7_Nd_0.3_)FeO_3_ for the BNFO matrix and (Fe_0.6_Bi_0.25_Nd_0.15_)FeO_3_ for the Fe-rich phase are shown in Fig. 4f,g with further information of simulations of HAADF images and Fe-*L*_2,3_ inelastic loss images given in Supporting Information. The simulations are consistent with the experimental observation and show the Fe-rich phase to have a stronger Fe signal than that of the BNFO matrix at all positions in the unit cell. The weak Fe signal from the Fe-rich, Fe/Bi/Nd mixed, A-site columns can be explained by the strong scattering of the heavier Bi/Nd-ions. It should be noted that the stronger O-*K* signal in the Fe-rich phase (Fig. 4e) is due to reduced Bi and Nd concentration on the A-site with respect to the matrix. It is concluded therefore that the phase is rich in Fe on the A-site with full Fe occupancy on the B-site, while the valence of the Fe ion remains to be elucidated.

**Figure 5:**
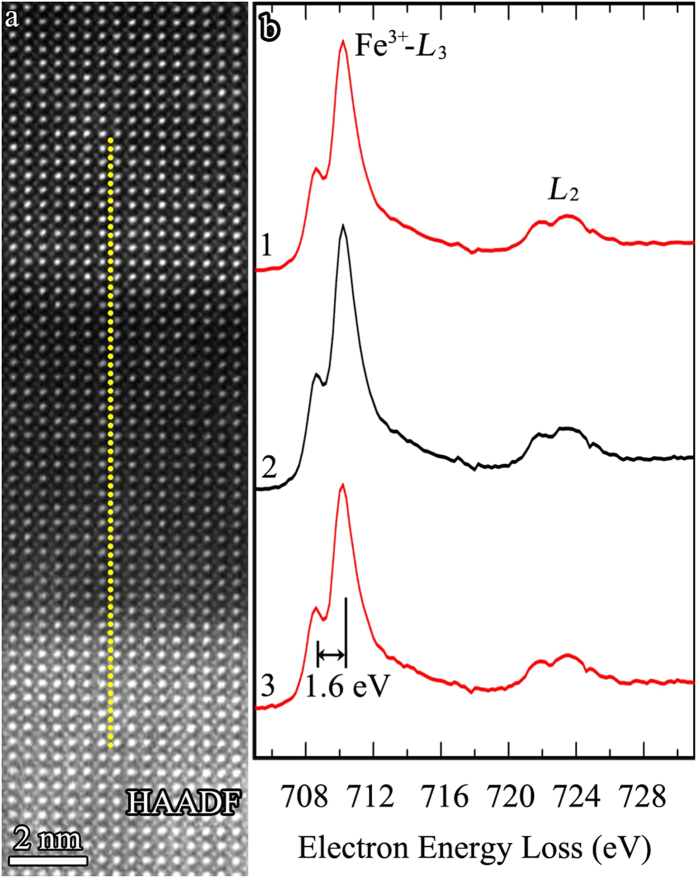
EELS map of the chemical valences of Fe-ions. (**a**) HAADF image showing a region containing the Fe-rich perovskite phase, the dotted yellow line illustrating the path of an EELS line-scan across the BNFO matrix and Fe-rich perovskite phase. (**b**) Background subtracted Fe-*L*_2,3_ spectra each averaged from 24 acquired spectra of upper matrix (spectrum 1), middle Fe-rich perovskite phase (spectrum 2) and bottom matrix (spectrum 3).

Figure 5a illustrates the position of an atomic resolution EELS line-scan across the Fe-rich phase to investigate possible fine structure changes in the Fe-*L*_2,3_ edges, which would be indicative of a valence change in the Fe-rich perovskite phase. Figure 5b shows three background subtracted spectra collected from the BNFO matrix (spectra 1 and 3) and from the Fe-rich phase (spectrum 2), each of which averaged from 24 acquired spectra to improve the signal-to-noise ratio. No difference is observed in the peak shape and position of the Fe-*L*_2,3_ edges between the three spectra, and importantly, no deviation is observed in spectrum 2 irrespective of where the average is extracted from. All the Fe-*L*_3_ edges are split, with a prepeak separated by 1.6 eV. Such a well-resolved splitting of the Fe-*L*_3_ edges is a characteristic of Fe^3+^ and inconsistent with Fe^2+^ or mixed valence, which, if present within the Fe-rich phase, would have contributed to a visible change in the fine structure of any spectrum acquired therein. This conclusively demonstrates dominant Fe^3+^ in both A- and B-sites, defining the base formula as (Fe_0.6_Bi_0.25_Nd_0.15_)^3+^ Fe^3+^O_3_.

**Figure 6:**
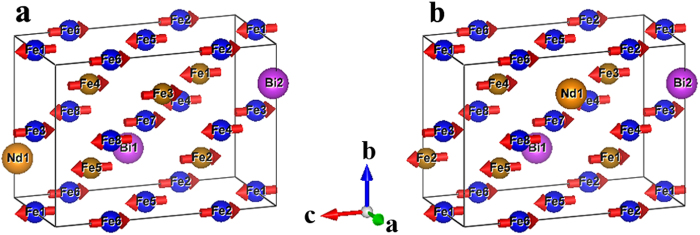
DFT calculated low energy state structures of Fe_2_O_3_-rich perovskite phase. (**a**,**b**) Two kinds of crystal structures with optimized magnetic configurations with a 40-atom supercell of Fe:Bi:Nd:Fe:O = 5: 2:1:8:24, in which Fe spins on both A (brown) and B (blue) sites are largely compensated but with weak residual ferrimagnetism. Oxygen atoms are omitted for the sake of clarity.

We carried out first-principles DFT calculations of the Fe_2_O_3_-rich perovskite nanophase using the Vienna ab initio simulation package (VASP)^22^ to better understand its potential physical properties. DFT calculations within the local spin density approximation (LSDA) plus the Hubbard parameter U (with U = 3.8 eV for Fe ions) were performed. To simulate the composition Fe:Bi:Nd:Fe:O = 12:5:3:20:60 from the above analysis, we adopted a 40-atom supercell with Fe:Bi:Nd:Fe:O = 5:2:1:8:24 to satisfy both periodicity and computational convenience. In the pseudocubic setting, our supercell has √2 × 2 × 2√2 five-atom formula units, and the computations have included oxygen octahedral tilting consistent with *Pnma* symmetry. B-site Fe ions that are nearest neighbours were imposed with opposite magnetic moments, consistent with the G-type antiferromagnetic order known to exist in BiFeO_3_. We used the projector augmented wave (PAW) method^23^ with an energy cutoff of 550 eV and a 1 × 2 × 2 k-point mesh. We first assigned 42 possible structure combinations with eight A sites containing 5 Fe, 2 Bi, and 1 Nd ions, and the total energy of each simulated structure was calculated from first principles. Then using the obtained low energy structures, we proceed to study the possible magnetic configurations by considering A site Fe ions (while keeping B site antiferromagnetic and treating A-site Bi and Nd as paramagnetic). Figure 6a,b show two kinds of crystal structures of a 40-atom supercell with optimized magnetic configurations. The total energy difference in these two structures is less than 1 meV. They are the lowest energy states from all the configurations. The opposite spin moments of neighbouring Fe atoms on A-site are largely compensated in a similar manner to those at the B site, but a small net magnetic moment results. Additionally, we built a 100-atom supercell Fe_12_Bi_5_Nd_3_Fe_20_O_60_ to perform the simulations, with the same conclusion as above. The results therefore suggest that the Fe_2_O_3_-rich perovskite phase is potentially ferrimagnetic.

Furthermore, we have also constructed the perovskite structures for Fe_2_O_3_, i.e. FeFeO_3_ with two Fe ions on A and B sites, by taking various oxygen octahedral tilting configurations into account. Nine 20-atom trial supercells (4 × 4 × 2 k-point mesh) were constructed with space groups of *Pm*-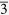
*m* (*a*^0^*a*^0^*a*^0^), *I*4/*mcm* (*a*^0^*a*^0^*c*^−^), *R*3*c* (*a*^−^*a*^−^*a*^−^), *I*4*cm* (*a*^0^*a*^0^*c*^−^), *Ima*2 (*a*^0^*b*^−^*b*^−^), *Imma* (*a*^0^*b*^−^*b*^−^), *P*4*mm* (*a*^0^*a*^0^*a*^0^), *Pnma* (*a*^−^*b*^−^*c*^+^), and *Pmc*2_1_ (*a*^0^*a*^0^*c*^+^). Similar to BiFeO_3_, we end up with the lowest energy state possessing *a*^*−*^*a*^*−*^*a*^*−*^ as in the *R*3*c or R*-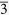
*c* space groups with the second lowest as *a*^−^*a*^−^*c*^+^ as in the space groups *Pna*2_1_ or *Pnma*. We note that the energy difference between the phases can be strongly dependent on the DFT functional. A detailed investigation to systematically search for stable phases of perovskite Fe_2_O_3_ is under way.

The observation of an Fe_2_O_3_-rich perovskite phase in Nd and Ti doped BiFeO_3_ thin films is of interest to the multiferroic community as it may affect the magnetic moment and additionally may be responsible for electrical breakdown in apparently single phase perovskite structured films. More importantly, the stabilisation of this phase at ambient pressure opens up the potential of new thin film materials with hitherto undefined properties but which should offer the possibility of multiple ferroic instabilities. We note that ε-Fe_2_O_3_ was recently reported to be ferroelectric and ferromagnetic at room temperature in ref. 24 and that weak ferromagnetism is observed in the films described here, possibly arising from the Fe_2_O_3_-rich perovskite phase rather than the antiferromagnetic matrix.

Further urgent research is thus required to fabricate single-phase films of these materials from clues revealed in this study. The Fe_2_O_3_-rich perovskite phase is favoured by low chamber pressure, potentially due to enhanced bombardment and re-sputtering of Bi during deposition which facilitate Fe incorporation on the A-site to accommodate Bi vacancies. On the other hand, Fe_2_O_3_-rich perovskite phase grows only in the upper region of the BNFO film, which suggests that the smaller lattice parameters of the BNFO matrix in the relaxed region (a_0_ ≈ 0.39 nm) facilitate the stabilization of Fe_2_O_3_-rich perovskite phase. The coherent growth suggests that epitaxial films of Fe_2_O_3_-rich perovskite phase could be fabricated on substrates which have a similar prototype perovskite lattice parameter to the Nd doped BiFeO_3_ matrix, such as DyScO_3_ (*a*_*0*_ = 0.394 nm) which are often utilised to fabricate coherently strained perovskites^18^. Furthermore, EDS suggests a formula (Fe_0.6_Bi_0.25_Nd_0.15_)FeO_3_, defining a likely PLD target composition.

In conclusion, evidence is presented of an unusual Fe_2_O_3_-rich perovskite nanophase coherent with the matrix (*a* ≈ 0.39 nm), that forms within epitaxial Ti doped (Bi_0.75_Nd_0.25_)FeO_3_ perovskite thin films deposited under conditions with high bombardment during pulsed laser deposition. The phase is stabilised by Bi and Nd ions on the A-site and the dominant valence is Fe^3+^, with an approximate formula (Fe_0.6_Bi_0.25_Nd_0.15_)^3+^ Fe^3+^O_3_. The incorporation of large ions such as Nd and Bi allows the Fe_2_O_3_-rich perovskite phase to form under coherent growth conditions, which suggests that incorporating large trivalent ions may therefore stabilise the high-pressure Fe_2_O_3_-rich perovskite phase. Preliminary DFT calculations suggest that the phase is potentially ferrimagnetic.

## Methods

### Film preparation

A (Bi_0.75_Nd_0.25_)(Fe_0.97_Ti_0.03_)O_3_ (BNFO) ceramic PLD target was prepared according to the process described in ref. 11. Films were deposited on (001) SrTiO_3_ (STO) substrates (CrysTec GmbH), using a home-built PLD system using a 248 nm KrF excimer laser (Coherent GmbH COMPexPro). Prior to deposition, STO substrates were ultrasonicated sequentially in acetone, ethanol, and isopropanol for 5 mins each, followed by 2 mins etch in BOE 10:1, rinsed with deionized water, and annealed at 1075 °C for 2 hrs. SrRuO_3_ (SRO) (SCI Engineered Materials) was first deposited as a bottom electrode on STO at a deposition temperature of 680 °C and oxygen partial pressure of 160 mTorr with the laser energy density of 2.9 J.cm^−2^ and a repetition rate of 10 Hz. The BNFO film was deposited on SRO/STO substrate at 650 °C and 50 mTorr oxygen partial pressure with the laser energy density of 4.2 J.cm^−2^ and repetition rate of 5 Hz. A 10% O_3_ /90% O_2_ ambient was used for all the depositions. The film was held at 450 °C for 20 min in 300 Torr 10% O_3_ in O_2_ ambient upon cooling for a post-deposition anneal.

### XRD analysis

X-ray diffraction analysis was carried out on a Philips Pro MRD to do *θ*–2*θ* and Phi scans to check the film crystallographic structure and epitaxial quality.

### Electrical measurements

BNFO film was etched with HCl solutions to expose the SrRuO_3_ bottom electrode in a corner. Photolithography (Clariant Corporation AZ5214E resist, Karl Suss MA/BA6 contact aligner) followed by sputter deposition (Kurt Lesker CMS-18) of 100 nm Pt was used to define top electrodes. Polarization vs. field measurements were performed using a Radiant RT-66 system to assess the ferroelectric hysteresis behaviour.

### Magnetic measurements

The sample was measured in a superconducting quantum interference device (SQUID) 5 Tesla magnetometer (Quantum Design 1802). The sample was centered at 305 K in a 9000 G field and field cooled in a 5 Tesla field to the target temperature. Field sweeps start at 5 T, sweep down to −5 T and then sweep up to 5 T.

### Atomic resolved characterization by aberration-corrected scanning transmission electron microscopy

TEM specimens were prepared with a dual beam FIB/SEM FEI Quanta 3D 200 machine. A double aberration corrected microscope JEM-Z3100F-R005 STEM/TEM operated at 300 keV (housed at the University of Sheffield) was used to screen the specimens with Fe-rich perovskite phase. Specimens were further thinned using FIB in low angle and low voltage conditions for reducing ion-beam damage. Quantitative EDS analysis was performed on a JEM-ARM200CF STEM/TEM equipped with a Bruker Quantax 60 mm^2^ SDD detector (housed at the University of Glasgow). The resulting data was quantified by use of a new spectral fitting approach (Elemental Quantification plugin for GMS 2.3, provided by Dr. Paul Thomas of Gatan UK Ltd.) using the Fe-*K*, Nd-*L* and Bi-*M* X-ray lines. The SuperSTEM facility’s Nion UltraSTEM microscope operated at 100 keV was used to collect the high angle annular dark field (HAADF) images, atomic resolution electron energy loss spectroscopy (EELS) spectrum-images and line-scan spectra. Some HAADF images were recorded as a stack of consecutive images by repetitive scanning of the same area rapidly at about 5 μs per pixel to minimize the effect of drift on individual images, followed by alignment and summation to create a high signal-to-noise ratio image with minimal distortion^25^. Unless otherwise specified, EELS spectrum-images were processed using principal component analysis (PCA) to remove random spectral noise^26^ and then the EELS spectra were integrated over a 75 eV window above the respective edge onset after power law background subtraction.

### Simulation of Fe-*L*_2,3_ inelastic loss images

Simulation of EELS images was carried out using the multislice method based on a frozen phonon model^19^,^20^,^21^. Atomic vibrational amplitudes for all structures were taken from the Bi_0.9_Nd_0.1_FeO_3_ *R*-phase structure in ref. 27. The 100 keV probe was assumed to be aberration-free, with a convergence semi-angle of 30.5 mrad and zero defocus. Spatial incoherence was included in the simulation by assuming an effective source size characterized by a Gaussian distribution of half-width at half-maximum 0.375 Å. An acceptance semi-angle of 36 mrad and energy integration window ΔE = 75 eV were taken for the Fe-*L*_2,3_ map simulations, using an isolated atom approximation. VESTA software was used to generate structure models^28^.

## Additional Information

**How to cite this article**: Zhang, H. *et al*. Stabilisation of Fe^2^O^3^-rich Perovskite Nanophase in Epitaxial Rare-earth Doped BiFeO^3^ Films. *Sci. Rep*. **5**, 13066; doi: 10.1038/srep13066 (2015).

## Supplementary Material

Supplementary Information
