# Supplementary material for: Stabilisation of Fe2O3-rich Perovskite Nanophase in Epitaxial Rare-earth Doped BiFeO3 Films
Source: Sci Rep. 2015 Aug 14;5:13066. doi: 10.1038/srep13066 (PMC4536528; doi:10.1038/srep13066)
Supplement: Supplementary Information [file srep13066-s1.pdf]

Supporting Information

Stabilisation of Fe_2O_3-rich Perovskite Nanophase in Epitaxial Rare-earth Doped $BiFeO_3$ Films

Huairuo Zhang[1*], Ian M. Reaney[1*], Daniel M. Marincel[2], Susan Trolier-McKinstry[2], Quentin M. Ramasse[3], Ian MacLaren[4], Scott D. Findlay[5], Robert D. Fraleigh[6], Ian M. Ross[7], Shunbo Hu[8], Wei Ren[8], W. Mark Rainforth[1]

[1]Department of Materials Science & Engineering, University of Sheffield, Sheffield S1 3JD, UK

[2]Department of Materials Science and Engineering and Materials Research Institute, The Pennsylvania State University, University Park, PA 16802, USA

[3]SuperSTEM Laboratory, STFC Daresbury Campus, Daresbury WA4 4AD, UK

[4]School of Physics and Astronomy, University of Glasgow, Glasgow G12 8QQ, UK

[5]School of Physics and Astronomy, Monash University, Clayton, Victoria 3800, Australia

[6]Department of Physics, The Pennsylvania State University, University Park, PA 16802, USA

[7]Kroto Centre for High Resolution Imaging & Analysis, Department of Electronic and Electric Engineering, University of Sheffield, Sheffield S1 3JD, UK

[8]Department of Physics, and International Center of Quantum and Molecular Structures, Shanghai University, Shanghai 200444, China

* Correspondence and requests for materials should be addressed to

H.Z. (Huairuo.Zhang@sheffield.ac.uk) or I.M.R. (I.M.Reaney@sheffield.ac.uk).

Simulations of HAADF images and Fe-$L_{2,3}$ inelastic loss images

Simulation of HAADF and EELS images was carried out using the multislice method based on a frozen phonon model[1-3]. Atomic vibrational amplitudes for all structures were taken from the $Bi_{0.9}Nd_{0.1}FeO_3$ *R*-phase structure in ref. [4]. The 100 keV probe was assumed to be aberration-free, with a convergence semi-angle of 30.5 mrad and zero defocus. The annular detector was assumed to have an inner radius of 86 mrad and an effective outer radius of 195 mrad, representative of measured experimental conditions. Spatial incoherence was included in the simulation by assuming an effective source size characterized by a Gaussian distribution of half-width at half-maximum 0.375 Å. An acceptance semi-angle of 36 mrad and energy integration window ΔE = 75 eV were taken for the Fe-$L_{2,3}$ map simulations, using an isolated atom approximation. VESTA software was used to generate structure models[5].

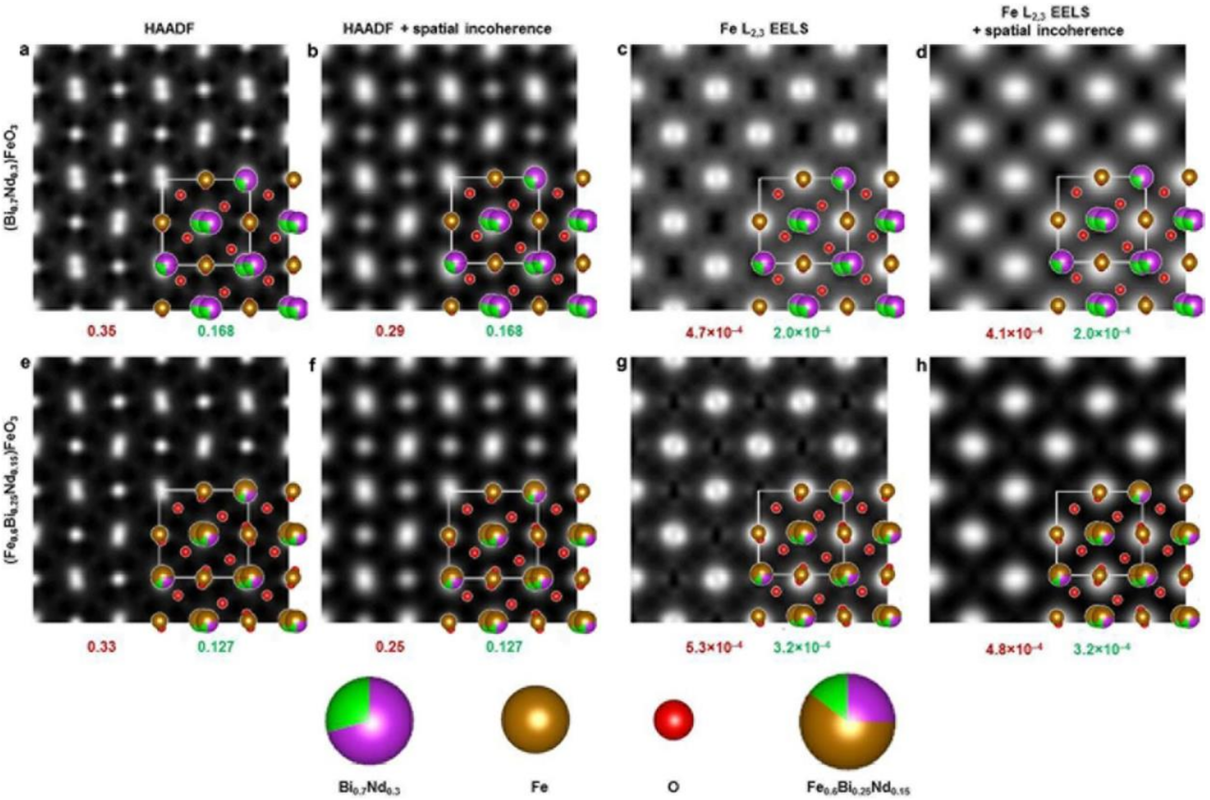

Figure S1. Simulations of HAADF images and Fe-$L_{2,3}$ inelastic loss images. (a – d) Simulations with a $(Bi_{0.7}Nd_{0.3})FeO_3$ structure model representing the BNFO matrix, and (e – h) with a $(Fe_{0.6}Bi_{0.25}Nd_{0.15})FeO_3$ structure model representing the Fe-rich perovskite phase. Simulations were carried out using a multislice method based on the frozen phonon model, with a thickness of 450 Å measured from the sampled EELS spectrum-imaging area. The red and green numbers below each image show the maximum and mean signal, respectively. Images S1d and S1h were taken for Figure 4f and 4g in the main text.

References:

1. Loane, R. F., Xu, P., Silcox, J. Thermal vibrations in convergent-beam electron diffraction. *Acta Cryst. A* **47,** 267-278 **(**1991).

2. Findlay, S. D., Oxley, M. P., Pennycook, S. J. & Allen, L. J. Modelling imaging based on core-loss spectroscopy in scanning transmission electron microscopy. *Ultramicroscopy* **104**, 126-140 (2005).
3. Bosman, M. *et al*. Two-Dimensional Mapping of Chemical Information at Atomic Resolution. *Phys. Rev. Lett.* **99**, 086102 (2007).
4. Levin, I. *et al*. Displacive Phase Transitions and Magnetic Structures in Nd-Substituted $BiFeO_3$. *Chem. Mater*. **23**, 2166-2175 (2011).
5. Momma, K. & Izumi, F. VESTA 3 for three-dimensional visualization of crystal, volumetric and morphology data. *J. Appl. Cryst*. **44**, 1272-1276 (2011).
